# Pretreatment prognostic factors of survival and late toxicities for patients with nasopharyngeal carcinoma treated by simultaneous integrated boost intensity-modulated radiotherapy

**DOI:** 10.1186/s13014-018-0990-5

**Published:** 2018-03-20

**Authors:** Yun-Hsuan Lin, Tai-Lin Huang, Chih-Yen Chien, Hui-Chun Chen, Hsuan-Chih Hsu, Eng-Yen Huang, Chong-Jong Wang, Yu-Jie Huang, Yu-Ming Wang, Chun-Chieh Huang, Shang-Yu Chou, Kuan-Cho Liao, Fu-Min Fang

**Affiliations:** 1 Department of Radiation Oncology, Kaohsiung Chang Gung Memorial Hospital and Chang Gung University College of Medicine, No. 123 Ta-Pei Rd., Niao Sung District, Kaohsiung, Taiwan; 2 Department of Hematology and Oncology, Kaohsiung Chang Gung Memorial Hospital and Chang Gung University College of Medicine, Kaohsiung, Taiwan; 3 Department of Otolaryngology, Kaohsiung Chang Gung Memorial Hospital and Chang Gung University College of Medicine, Kaohsiung, Taiwan

**Keywords:** Nasopharyngeal carcinoma, Simultaneous integrated boost intensity-modulated radiation therapy, Hematologic inflammatory marker, Epstein-Barr virus DNA, Gross tumor volume

## Abstract

**Background:**

To scrutinize the pretreatment prognosticators on survival and late toxicities in a homogenous cohort of nasopharyngeal carcinoma (NPC) patients treated by simultaneous integrated boost intensity-modulated radiation therapy (SIB-IMRT).

**Methods:**

A total of 219 non-distant metastatic NPC patients consecutively treated by SIB-IMRT at a single institute were collected. The pretreatment factors including the socio-demographic variables, TNM stages, gross tumor volume (GTV), Epstein-Barr virus (EBV)-DNA, and hematologic inflammatory markers were analyzed. Cox model was used to screen the prognostic factors of late toxicities and four survival outcomes including locoregional relapse-free survival (LRRFS), distant metastasis-free survival (DMFS), failure-free survival (FFS), and overall survival (OS).

**Results:**

Statistically significant inter-correlations were observed between the values of EBV-DNA, some hematologic inflammatory markers, GTV, and N classification. The 5-year LRRFS, DMFS, FFS, and OS rates were 87.9%, 89.4%, 79.4%, and 81.3%, respectively. Multivariate analysis revealed that advanced N classification (N2–3 vs. N0–1) remained the only significant negative prognosticator for all the four survival outcomes. An increased monocyte percentage and a decreased lymphocyte-to-monocyte ratio were significantly associated with poorer FFS and OS, respectively. Larger GTV was observed to be predictive of poorer LRRFS. Patients with T3–4 (HR: 3.5, 95% CI: 1.0–12.1, *p* = 0.048) or higher GTV (HR: 1.006, 95% CI: 1.001–1.011, *p* = 0.027) were associated with higher incidence of radiation neuropathy.

**Conclusion:**

N classification remains the most significant survival predictor for NPC patients treated by SIB-IMRT after adjusting these biomarkers. GTV impacts not only on locoregional control but also radiation neuropathy.

## Background

Nasopharyngeal carcinoma (NPC) is endemic in Taiwan, Southern China, and Southeast Asia, with annual incidence of 15–50 cases per 100,000 [1]. Radiation therapy (RT) is essential to successful treatment of NPC, owing to the location and radio-sensitivity of the disease. In consideration of the proximity of the nasopharynx to many critical organs, it is challenging to provide adequate dose to the gross tumor and nodes while simultaneous sparing those surrounding critical organs. Intensity-modulated radiotherapy (IMRT) employs inverse planning algorithms and iterative computer-driven optimization to generate treatment fields with varying beam intensity, which enables the simultaneous integrated boost (SIB) to different target volumes with different dose levels. In contrast to IMRT with two-phase shrinkage technique, SIB-IMRT has been shown to be more advanced in dosimetric outcome for NPC and become the standard treatment in the radiation oncology community [2].

With the use of the IMRT alone or with the combination of chemotherapy (C/T) in the treatment of NPC, the overall survival rate of NPC has upwardly revised to exceed 80% [3–6]. The failure patterns of NPC have been totally different compared to the reports in the conventional two-dimensional RT (2DRT) era, and the divergence of survival rates of different clinical stages has greatly reduced [4, 5]. Thus, the TNM staging system has been criticized to insufficiently predict the prognosis of NPC, and more and more studies have been focused on the investigation of some novel pretreatment factors, which include plasma Epstein-Barr virus (EBV)-DNA, hematologic inflammatory markers, and gross tumor volume (GTV), to additionally increase the prognostic accuracy [7–18]. However, most of these survival prognosticators were individually analyzed from NPC patients treated with heterogeneous components of RT techniques [14–16, 19, 20].

Besides the failure pattern and survival outcome, the severity of late toxicities has also greatly changed with the evolution of RT techniques. It has been well established that the dosimetric superiority of IMRT over 2DRT could transfer to the reduction of late toxicities and thereby improve the quality of life for NPC survivors [21]. As far as we know, the determinants of late toxicities for NPC patients were mostly focused on the treatment-related variables in the literature, and the pretreatment factors, especially the biological factors, were seldom explored.

On the basis of this premise, we conducted the study in a homogenous cohort of NPC patients treated by SIB-IMRT, with the main goal to comprehensively scrutinize the impact of pretreatment factors, including socio-demographic variables, TNM stages, EBV-DNA, hematologic inflammatory markers, and GTV, on the survival outcomes and late toxicity.

## Methods

### Patient characteristics

Table 1 outlines the patients’ socio-demographic and clinical characteristics. Altogether, we analyzed 219 consecutive patients with previously untreated, biopsy-proven non-distant metastatic NPC. The median age at the time of the diagnosis of NPC was 52 years old, ranged from 15 up to 87 years old. There were 161 males and 58 females. Only 3 patients had WHO type I NPC, but as many as 216 patients had WHO type II NPC. The American Joint Committee on Cancer (AJCC) 7th edition stage distribution was I: 12.3%, II: 20.6%, III: 30.6%, and IVA-IVB: 36.5%, respectively. All patients were treated between August 2009 and December 2014 at Kaohsiung Chang Gung Memorial Hospital. This study was conducted in compliance with the institutional policy regarding the protection of patients’ private information and was approved by the Institutional review board/Ethics committee (IRB/EC) of Kaohsiung Chang Gung Memorial Hospital. Informed consent was obtained from all patients.

**Table 1:** Patient characteristics (*n* = 219)

Variable	Number	Percent
Age, range (median)	15–87 (52)	
≤ 50 year	97	44.3
> 50 year	122	55.7
Gender		
Male	161	73.5
Female	58	26.5
Educational level		
≤ 12 year	165	75.3
> 12 year	54	24.7
Marital status		
With spouse	160	73.1
Without spouse	59	26.9
Smoking history		
Yes	92	42.0
No	127	58.0
Charlson comorbidity index		
0	125	57.1
≥ 1	94	42.9
Body mass index		
< 23	62	28.8
≥ 23	157	71.2
WHO histology		
Type I	3	1.4
Type IIA	98	44.7
Type IIB	118	53.9
Clinical stage^a^		
I	27	12.3
II	45	20.6
III	67	30.6
IVA-B	80	36.5
T classification^a^		
T1	98	44.7
T2	35	16.0
T3	48	21.9
T4	38	17.4
N classification^a^		
N0	37	16.9
N1	72	32.9
N2	64	29.2
N3	46	21.0
Chemotherapy		
Concurrent chemotherapy	192	87.7
Adjuvant chemotherapy	144	65.8

*WHO* World Health Organization

^a^According to the AJCC 7th staging system

### Pretreatment evaluation

The routine pretreatment evaluation consisted of complete history taking, physical and nasopharyngoscopy examinations. Hematologic inflammatory markers, including percentage of neutrophil, percentage of lymphocyte, percentage of monocyte, platelet count, neutrophil-to-lymphocyte ratio (N/L ratio), lymphocyte-to-monocyte ratio (L/M ratio), platelet-to-lymphocyte ratio (P/L ratio), were obtained before the beginning of the course of treatment. All patients had no evidence of acute infection and hematologic disorders, indicating that the hematologic inflammatory markers could represent the normal baseline values. Pretreatment plasma EBV-DNA was measured by real-time quantitative polymerase chain reaction. Magnetic resonance imaging (MRI) of the head and neck was required. Additional imaging studies to evaluate the extent of disease included the chest radiography, liver ultrasonography, and bone scan. Whole body positron emission tomography-computed tomography (PET-CT) was optional.

### Treatment methods

The details of the SIB-IMRT technique for NPC in the institute have been reported previously [22]. The planning system Pinnacle^3^ (version 9.2, Philips) was used. IMRT was delivered by step-and-shoot 7-field technique or dual arc technique. Computerized optimization was used with fusion of MRI and/or PET with treatment planning CT images, when possible, to accurately delineate the GTV, which included the primary disease and nodes greater than 1 cm in diameter or nodes with necrotic centers. The values of GTV in the study were calculated by the treatment planning system. Three different dose levels of clinical target volumes (CTVs) were created. The high dose level of CTV (CTV-H) was defined as the GTV with an isotropic extension of 5 mm. The middle dose level of CTV (CTV-M) covered the CTV-H plus the areas at risk for microscopic involvement, including the entire nasopharynx, posterior third of nasal cavity and maxillary sinus, pterygoid plate, parapharyngeal space, retropharyngeal lymph nodes, clivus, skull base, inferior sphenoid sinus and bilateral upper neck nodes. The low dose level of CTV (CTV-L) included the CTV-M plus bilateral lower neck nodes. To account for organ motion and daily treatment set-up uncertainties, planning target volumes (PTVs) were generated with additional margins of 3 to 5 mm to each of the CTVs. The prescribed dose and fractionation for PTV-H, PTV-M, and PTV-L were 69.96 or 69.30 Gy, 59.40 Gy, and 52.80 Gy in 33 fractions, respectively. The delineation of the organs at risk (OARs) and constraints of the dosage applied to OARs were under the framework of Radiation Therapy Oncology Group (RTOG) 0225 protocol [3].

Patients with stage II to IVB received concurrent C/T with weekly cisplatin 30–40 mg/m^2^ administered during the SIB-IMRT courses. Adjuvant C/T with cisplatin 70–80 mg/m^2^ on day 1 and 5-fluorouracil 700–800 mg/m^2^/d on day 1–4 administered every 3–4 weeks was given for 1–4 cycles to those patients with residual tumor or receiving inadequate doses of cisplatin during the course of RT. Dose modification of the C/T regimens was determined according to the judgment of patients’ conditions by each medical oncologist [23]. The combination of C/T was performed in 87.8% of patients at concurrent, and 65.8% at adjuvant phase, respectively.

### Assessment of late toxicity

The severities of six late toxicities (xerostomia, hearing impairment, chronic otitis media, dysphagia, neck fibrosis, and neuropathy) that were evaluated prospectively in the radiation oncology medical records at each visit were collected. Maximal late toxicities that persisted or occurred during the period of 3 months after RT to the date of last visit were recorded and based on the criteria of Common Terminology Criteria for Adverse Events version 4.03.

### Follow-up

Patients were regularly followed up after RT until death or their last follow-up appointment. They were scheduled to visit the clinics at 3-month, and 4- to 6-month intervals in the first two, and third to fifth years, respectively. The median followed-up months were 42.4 months (range, 2 to 83.7 months). Physical and nasopharyngoscopy examinations were routinely performed at every visit. Head and neck MRI scans were performed within 2 months after RT and annually within the first 5 years after RT or when there were clinical indications. Locoregional failure was determined based on pathologic diagnosis or progressive deterioration shown on consecutive image studies. To identify distant metastases, patients were scrutinized by chest X-ray yearly and by abdominal sonogram or bone scan whenever indicated.

### Statistical analysis

The primary endpoint was to scrutinize the pretreatment prognostic factors of survival and late toxicities, and the secondary endpoint was to analyze the inter-correlations between the pretreatment variables. The Pearson correlation was used to assess the inter-correlations between the pretreatment variables. The duration of survival was calculated from the last day of RT. Patients alive on the last day of follow-up were censored. Survival curves including the locoregional relapse-free survival (LRRFS), distant metastasis-free survival (DMFS), failure-free survival (FFS), and overall survival (OS) were estimated by the Kaplan-Meier method. The log rank test was used to estimate the statistical significance of differences between survival curves. The Cox proportional hazards regression model was used for univariate and multivariate analysis. A *p*-value of less than 0.05 was considered to indicate statistical significance. The receiver operating characteristic (ROC) curve analysis was applied to evaluate the cutoff point of the continuous value of the variable, which revealed to be a significant predictor after analysis. The Microsoft Statistical Package for Social Sciences version 20.0 software (SPSS, Chicago, IL) was used for statistical processing.

## Results

### Inter-correlations of EBV-DNA, hematologic inflammatory markers, T&N classification, and GTV

Statistically significant inter-correlations were observed between the values of EBV-DNA, some hematologic inflammatory markers, GTV, and N classification (Table 2). Regarding the EBV-DNA and hematologic inflammatory markers, the value of EBV-DNA was observed to be significantly positively correlated with the percentage of monocyte (γ:0.28, *p* < 0.001), platelet count (γ:0.20, *p* < 0.01) and P/L ratio (γ:0.32, *p* < 0.001), but negatively correlated with the percentage of lymphocyte (γ:-0.27, *p* < 0.001) and L/M ratio (γ:-0.29, *p* < 0.001). Significant correlations were also observed between the value of EBV-DNA with N classification (γ: 0.31, *p* < 0.001) and GTV (γ: 0.39, *p* < 0.001). Regarding GTV and hematologic inflammatory markers, GTV was observed to be significantly positively correlated with the percentage of monocyte (γ:0.18, *p* < 0.01), platelet count (γ:0.27, *p* < 0.001) and P/L ratio (γ:0.18, *p* < 0.01), but negatively correlated with L/M ratio (γ:-0.17, *p* < 0.01).

**Table 2:** Inter-correlations of EBV-DNA, hematologic inflammatory markers, T&N classification, and GTV

		EBV-DNA	T classification^a^	N classification^a^	GTV
	Median (range)	γ	γ	γ	γ
Neutrophil, %	63.1 (31.7–86.1)	0.09	0.10	−0.04	−0.07
Lymphocyte, %	28.4 (10.0–51.3)	−0.27^**^	0.11	0.03	−0.01
Monocyte, %	5.6 (0.8–11.7)	0.28^**^	0.10	0.01	0.18^*^
Platelet, 10^9^/l	236 (112–498)	0.20^*^	0.07	0.07	0.27^**^
N/L ratio	2.21 (0.62–8.61)	0.14	−0.08	−0.04	−0.02
L/M ratio	5.04 (1.25–21.25)	−0.29^**^	−0.05	0.05	−0.17^*^
P/L ratio	8.43 (3.14–37.60)	0.32^**^	−0.01	0.01	0.18^*^
EBV-DNA, copies/ml	106 (0–94,920)	–	0.04	0.31^**^	0.39^**^

*N/L ratio* neutrophil to lymphocyte ratio, *L/M ratio* lymphocyte-to-monocyte ratio, *P/L ratio* platelet to lymphocyte ratio, *EBV* Epstein-Barr virus, *GTV* gross tumor volume

^*^p < 0.01, ^**^ *p* < 0.001, γ: correlation coefficient

^a^According to the AJCC 7th staging system

### Survival outcomes

There were 40 patients who experienced treatment failures, including local recurrence in 15 patients, regional recurrence in 14 patients, and distant metastasis in 24 patients. The most frequently involved metastatic sites were bone (17 patients), liver (17 patients), and lung (13 patients). After treatment, only one patient, with initial T3 N2 disease, had detectable post-treatment EBV-DNA. For the 40 patients who experienced treatment failure, 34 patients had detectable EBV-DNA at the time of relapse. At the last follow-up, there were 35 patients died, and seven of them died from locoregional recurrences, 15 from distant metastases, eight from second primary malignancies or the other medical co-morbidities, and five from unknown causes. The resulting 5-year LRRFS, DMFS, FFS, and OS rates were 87.9%, 89.4%, 79.4%, and 81.3%, respectively.

### Cox models of pretreatment predictors for survival

**Fig. 1:**
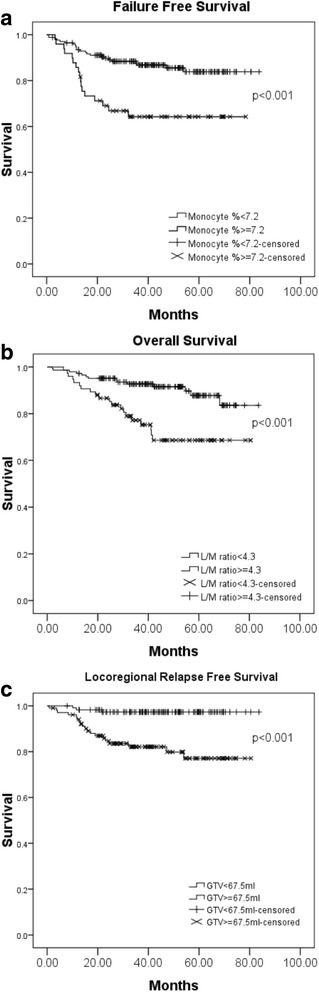
Survival comparisons of NPC patients by different pretreatment biomarkers based on the cutoff point through ROC curve analysis: (**a**) Failure-free survival for those with monocyte percentage < 7.2% vs. ≥ 7.2%; (**b**) Overall survival for those with L/M ratio ≥ 4.3 vs. < 4.3; (**c**) Locoregional relapse-free survival for those with gross tumor volume < 67.5 ml vs. ≥ 67.5 ml

The Cox models of univariate and multivariate analysis for pretreatment survival predictors with statistical significance are shown in Table 3. Multivariate analysis revealed that advanced N classification (N2–3 vs. N0–1) remained the only significant negative prognosticator for all the four survival outcomes. The 5-year LRRFS, DMFS, FFS, and OS rates for those with N0–1 were 97.1%, 94.9%, 90.9%, and 92.8% compared with 78.0%, 83.7%, 67.9%, and 69.4% for those with N2–3 (all *p* values < 0.001), respectively. The significant predictive value of pretreatment plasma EBV-DNA level (continuous or cutoff level at 1500 copies/ml) was observed for FFS or OS in univariate analysis but not observed for any of the four survival results after multivariate analysis. As regards the hematologic inflammatory markers, an increased monocyte percentage was significantly associated with poorer FFS (HR: 1.261, 95% CI: 1.057–1.503, *p* = 0.010) and a decreased L/M ratio associated with poorer OS (HR: 0.676, 95% CI: 0.488–0.936, *p* = 0.018). Through ROC curve analysis, the cutoff point was 7.2 for monocyte percentage and 4.3 for L/M ratio, respectively. The 5-year FFS rate was 83.8% for those with monocyte percentage < 7.2, compared with 64.2% for those with monocyte percentage ≥ 7.2 (*p* < 0.001, Fig. 1a). On the other hand, the 5-year OS rate was 68.6% for those with L/M ratio < 4.3, compared with 87.8% for those with L/M ratio ≥ 4.3 (*p* = 0.001, Fig. 1b). Meanwhile, larger GTV was observed to be predictive of poorer LRRFS. A 10 ml increase of GTV was associated with a 5% (95% CI, 1% to 10%, *p* = 0.023) increment in the likelihood of locoregional failure. Through ROC curve analysis, the cutoff point of GTV was 67.5 ml. The 5-year LRRFS rate was 97.3% for those with GTV < 67.5 ml, compared with 77.1% for those with GTV ≥ 67.5 ml (*p* < 0.001, Fig. 1c).

**Table 3:** Cox models of predictors for survival results

Variables	Univariate			Multivariate		
	HR	95% CI	*p*	HR	95% CI	*p*
LRRFS						
T classification^a^ (T1–2 vs T3–4)	2.4	1.1–5.7	0.039	1.4	0.6–3.5	0.490
N classification^a^ (N0–1 vs N2–3)	7.1	2.1–24.2	0.002	5.9	1.6–21.9	0.009
Gross tumor volume (continuous)	1.008	1.004–1.013	< 0.001	1.005	1.001–1.010	0.013
Lymphocyte, % (continuous)	0.956	0.916–0.999	0.045	0.971	0.926–1.019	0.235
Monocyte, % (continuous)	1.322	1.073–1.629	0.009	1.210	0.997–1.041	0.061
DMFS						
Gender (Male vs Female)	0.2	0.1–0.9	0.041	0.2	0.1–1.2	0.076
N classification^a^ (N0–1 vs N2–3)	3.8	1.4–10.3	0.009	3.7	1.3–9.8	0.012
Gross tumor volume (continuous)	1.006	1.002–1.011	0.005	1.003	0.998–1.008	0.223
FFS						
Gender (Male vs Female)	0.3	0.1–0.8	0.016	0.4	0.1–1.2	0.104
T classification^a^ (T1–2 vs T3–4)	1.9	1.0–3.5	0.048	1.4	0.7–2.7	0.369
N classification^a^ (N0–1 vs N2–3)	4.0	1.9–8.5	< 0.001	3.9	1.6–9.0	0.001
Gross tumor volume (continuous)	1.007	1.004–1.010	< 0.001	1.002	0.998–1.006	0.270
EBV DNA (< 1500 vs ≥ 1500 copies/ml)	3.7	1.1–9.2	0.031	1.2	0.3–3.6	0.536
Monocyte, % (continuous)	1.255	1.072–1.470	0.005	1.261	1.057–1.503	0.010
OS						
Age (continuous)	1.028	1.000–1.059	0.044	1.041	1.001–1.082	0.046
Educational level (≤ 12 vs > 12 year)	0.3	0.1–0.9	0.040	0.4	0.1–1.9	0.259
Charlson Comorbidity Index (0 vs ≥ 1)	2.0	1.0–4.0	0.043	2.3	0.9–6.2	0.098
T classification^a^ (T1–2 vs T3–4)	2.5	1.3–5.0	0.008	2.3	0.9–6.0	0.100
N classification^a^ (N0–1 vs N2–3)	3.8	1.7–8.3	< 0.001	7.3	1.8–29.0	0.005
Gross tumor volume (continuous)	1.007	1.004–1.011	0.001	1.002	0.995–1.008	0.639
EBV DNA (< 1500 vs ≥ 1500 copies/ml)	4.2	1.8–10.0	0.001	1.5	0.5–4.3	0.426
L/M ratio (continuous)	0.788	0.649–0.957	0.018	0.676	0.488–0.936	0.018

*LRRFS* locoregional relapse-free survival, *DMFS* distant metastasis-free survival, *FFS* failure-free survival, *OS* overall survival, *L/M ratio* lymphocyte-to-monocyte ratio, *EBV* Epstein-Barr virus, *HR* hazard ratio, *CI* confidence interval

^a^According to the AJCC 7th staging system

### Late toxicity

Xerostomia, hearing impairment, chronic otitis media, dysphagia, and neck fibrosis were the common late toxicities concerned. The distributions of the five late toxicities with grade 2 or more were 73 (33.3%) in chronic otitis media, followed by 64 (29.2%) in xerostomia, 62 (28.3%) in hearing impairment, 51 (23.3%) in dysphagia, and 8 (3.7%) in neck fibrosis, respectively (Table 4). The 5-year cumulative incidence rate of developing at least one of the five late toxicities with grade 3 or more was 4.4%. In addition, radiation neuropathy was observed in 14 (6.4%) patients, including temporal lobe necrosis in 10 (4.6%) patients, and cranial nerve palsy in 4 (1.8%) patients. Temporal lobe necrosis was diagnosed based on the finding from MRI. All the radiation neuropathies were mild and do not interfere with the activity of patients’ daily life (grade 1). The 5-year cumulative incidence rate of radiation neuropathy was 8.1%.

**Table 4:** Frequency of late toxicity

	Grade 0	Grade 1	Grade 2	Grade 3	Grade 4
	N (%)	N (%)	N (%)	N (%)	N (%)
Xerostomia	48 (21.9)	107 (48.9)	64 (29.2)	0 (0)	0 (0)
Hearing impairment	75 (34.2)	82 (37.5)	51 (23.3)	4 (1.8)	7 (3.2)
Chronic otitis media	74 (33.8)	72 (32.9)	73 (33.3)	0 (0)	0 (0)
Dysphagia	94 (42.9)	74 (33.8)	50 (22.8)	1 (0.5)	0 (0)
Neck fibrosis	140 (63.9)	71 (32.4)	6 (2.7)	2 (1.0)	0 (0)
Radiation neuropathy	205 (93.6)	14 (6.4)	0 (0)	0 (0)	0 (0)
Temporal lobe necrosis	209 (95.4)	10 (4.6)	0 (0)	0 (0)	0 (0)
Cranial nerve palsy	215 (98.2)	4 (1.8)	0 (0)	0 (0)	0 (0)

Graded according to the CTCAE Version 4.03

### Cox model of pretreatment predictors for late toxicities

**Fig. 2:**
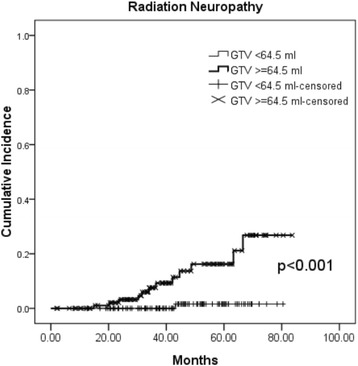
Cumulative incidence rate of radiation neuropathy for those with gross tumor volume < 64.5 ml vs. ≥ 64.5 ml

We could not find any pretreatment factor to be significantly associated with the development of the five late toxicities with grade 3 or more. On the contrast, T classification and GTV were observed to be significantly associated the occurrence of radiation neuropathy in univariate or multivariate analysis. The Cox model as shown in Table 5 revealed patients with T3–4 (HR: 3.5, 95% CI: 1.0–12.1, *p* = 0.048) or higher GTV (HR: 1.006, 95% CI: 1.001–1.011, *p* = 0.027) was predictive of radiation neuropathy. Through ROC curve analysis, the cutoff point of GTV was 64.5 ml. The 5-year cumulative incidence rate of radiation neuropathy was 1.6% for those with GTV < 64.5 ml compared with 16.2% for those with GTV ≥ 64.5 ml (*p* < 0.001, Fig. 2).

**Table 5:** Cox model of predictors for radiation neuropathy

Variables	Univariate			Multivariate		
	HR	95% CI	*p*	HR	95% CI	*p*
Radiation neuropathy						
T classification^a^ (T1–2 vs T3–4)	4.7	1.4–15.3	0.010	3.5	1.0–12.1	0.048
Gross tumor volume (continuous)	1.007	1.002–1.013	0.004	1.006	1.001–1.011	0.027

*HR* hazard ratio, *CI* confidence interval

^a^According to the AJCC 7th staging system

## Discussion

To the best of our knowledge, the current study is the first to comprehensively explore the associations and predictive value of pretreatment factors including socio-demographic variables, clinical stages, EBV-DNA, hematologic inflammatory markers, and GTV, in a large-scale homogenous NPC cohort treated by SIB-IMRT. Significant inter-correlations between EBV-DNA, some hematologic inflammatory markers, GTV and N classification were observed, and N classification remains the most important survival predictor for NPC patients after adjusting these covariates.

SIB-IMRT technique owns the advantage of increasing the dose per fraction to the tumor area with reduction of overall treatment time, which leads to increased biologically equivalent dose and theoretically decreased tumor repopulation and increased tumor control [2]. Currently, with the use of SIB-IMRT, the 5-year locoregional control of NPC has upwardly approached to 85–90% [3–6]. The discrimination of locoregional control based on different clinical stages has remarkably reduced, thus, it is not surprising that T classification was not observed to be an independent survival predictor in our study or other reports [4, 5]. On the contrary, distant metastasis remained the major failure pattern, and advanced N classification was observed to be the only significant negative prognosticator for all the four survival outcomes, although the combinations of systemic C/T with IMRT were extensively applied in cases with locally advanced stages.

It remains unclear as regards the associations of EBV-DNA, hematologic inflammatory markers, and tumor burden. EBV infection contributes the most to the carcinogenesis of NPC, and EBV-infected NPC cells harness immune cells and facilitate a protumorigenic inflammatory tumor microenvironment [9]. Some basic researches have noted the relationship between EBV infection and the activation or inhibition of these blood cells [24, 25]. Higher EBV-DNA was observed to be associated with more advanced clinical stage or larger tumor volume [19, 20], and persistent elevation of post-treatment EBV-DNA was associated with a higher chance of relapse and death [26]. Regarding the hematologic inflammatory markers, Gao et al. observed the positive association between platelet count and T classification; Chua et al. observed the positive association of N/L ratio with advanced T classification, N classification, and high EBV-DNA; and Jiang et al. observed the positive association of P/L ratio with clinical stage, T classification, and EBV-DNA [14–16]. In the study, we also demonstrate the links among the EBV-DNA level, severity of inflammation (positively correlated with monocyte and platelet but negatively correlated with lymphocyte) and tumor burden, although the actual mechanism has not yet been thoroughly investigated currently. In this cohort, the survival predictability of pretreatment EBV-DNA level was not observed after multivariate analysis, which might indicate the survival predictability of EBV-DNA might be diluted by these inter-correlated variables.

Inflammation has long been associated with the development of cancers, and chronic systemic inflammatory response has been clearly implicated in the progressive process and subsequent poor outcomes of cancer patients [10, 27]. However, there still exists controversy that which pretreatment hematologic inflammatory marker is a reliable survival predictor in cancer patients. Neutrophil, lymphocyte, monocyte, platelet, N/L ratio, L/M ratio, or P/L ratio has been reported to be independent survival predictors for NPC patients in different studies [9–16]. The survival predictability of N/L ratio was not observed in the study, though its survival predictability has been commonly revealed in a variety of cancers [9, 11, 13, 27]. On the contrast, we observed that an increased monocyte percentage and a decreased L/M ratio were significantly associated with poorer FFS and OS, respectively. Lymphocytes and monocytes are key immune cells in the inflammatory response, and have been reported to be independently associated with the prognosis of various malignancies, such as gastric cancer, acute lymphoblastic leukemia, lymphoma, hepatocellular carcinoma, and NPC [10, 28–31].

GTV is a direct indicator of tumor burden. Diseases with larger tumor burden afford a favorable environment for proliferation of hypoxic cells and G0 cells, and thus result in higher radioresistance [18]. Accurate measurement of GTV was difficult under conventional 2DRT, but has become easily available with the advent of IMRT. Several studies have reported the influence of GTV on locoreginal control and survival for NPC patients [17, 18]. In our cohort, GTV was observed to be significantly predictive of locoregional control only. It is worth noting that, in the current study, the GTV, which is the target subjected to receive the highest dose in the routine SIB-IMRT planning, included both primary gross tumor and enlarged neck nodes that meet the criteria, rather than solely the primary tumor or in combination with enlarge retropharyngeal lymph nodes, as defined in the other studies [17, 18]. Thereby, its survival predictability might be also diluted with the adjustment of the N classification in the statistical process.

In addition to parotid sparing, IMRT provides dosimetric benefits in the critical structures of skull base, temporal lobe, middle ear, and cochlea for NPC planning [32]. The incidence rate of developing severe (grade 3 or more) late toxicities in the previously commonly concerned items, such as xerostomia, hearing impairment, chronic otitis media, dysphagia and neck fibrosis, has remarkably declined with SIB-IMRT (4.4% in our series). Not surprisingly, we could not detect any significant pretreatment variable to predict these toxicities. As regards the radiation neuropathy, though the incidence was also largely decreased in the era of SIB-IMRT, temporal lobe necrosis remains an issue to be concerned. A study by Zeng et al. showed that, with the application of IMRT in patients with NPC, the incidence of most radiation-related neurological complications were reduced, except for temporal lobe necrosis [33]. Although the severities of temporal lobe necrosis and cranial nerve palsy were mild in our patients with limited follow-up, it might become progressive and affected the quality of life of NPC patients with long-term survival [21]. The determinants of radiation neuropathy in previous reports included T classification, total RT dose, fraction size, overall treatment time, and receiving C/T. These patients analyzed received heterogeneous RT techniques, varied from conventional 2DRT, 3D conformal RT, to IMRT [33–35]. On the contrast, with a consistent technique of SIB-IMRT for our patients, we observed the pre-treatment variables of advanced T classification and larger GTV were significant independent predictors for the occurrence of radiation neuropathy. There are growing reports announcing the therapeutic benefits in dosimetry, tumor control or patients’ quality of life if re-planning during the treatment course of SIB-IMRT was conducted, though its optimal criteria has yet to be determined [36]. To reduce the radiation neuropathy in those NPC patients with advanced T stage and large GTV remains big challenge, however, the adaptive technique with re-planning for those with remarkable reduction of GTV during SIB-IMRT deserves further evaluation.

Several limitations exist in the study. First, longer follow-up period might be needed to detect all the possible events. Second, a universally recognized measurement of plasma EBV-DNA has not been established, which might yield an institutional bias [37]. Third, the study cohort was exclusively from a single institution to ensure the homogeneity of the sample, therefore, our results should be interpreted with caution when extrapolating to patients in other regions.

## Conclusion

In conclusion, N classification remains the most powerful survival predictor for NPC patients treated by SIB-IMRT after adjusting these biomarkers. The association between EBV-DNA, hematologic inflammatory markers and tumor burden deserves further exploration. GTV impacts not only on locoregional control but also radiation neuropathy.

## Abbreviations

2DRT: Two-dimensional RT
AJCC: American Joint Committee on Cancer
C/T: Chemotherapy
CTV: Clinical target volume
DMFS: Distant metastasis-free survival
EBV: Epstein-Barr virus
FFS: Failure-free survival
GTV: Gross tumor volume
IRB/EC: Institutional review board/Ethics committee
L/M ratio: Lymphocyte-to-monocyte ratio
LRRFS: Locoregional relapse-free survival
MRI: Magnetic resonance imaging
N/L ratio: Neutrophil-to-lymphocyte ratio
NPC: Nasopharyngeal carcinoma
OAR: Organ at risk
OS: Overall survival
P/L ratio: Platelet-to-lymphocyte ratio
PET-CT: Positron emission tomography-computed tomography
PTV: Planning target volume
ROC: Receiver operating characteristic
RT: Radiation therapy
RTOG: Radiation Therapy Oncology Group
SIB-IMRT: Simultaneous integrated boost intensity-modulated radiation therapy
